# Effects of *Clostridium butyricum* on Growth Performance, Gut Microbiota and Intestinal Barrier Function of Broilers

**DOI:** 10.3389/fmicb.2021.777456

**Published:** 2021-12-08

**Authors:** Wenjia Li, Bin Xu, Linyi Wang, Quanyou Sun, Wen Deng, Fengxian Wei, Huihui Ma, Chen Fu, Gaili Wang, Shaoyu Li

**Affiliations:** Institute of Animal Husbandry and Veterinary Science, Henan Academy of Agricultural Sciences, Zhengzhou, China

**Keywords:** broilers, *Clostridium butyricum*, growth, gut microbiota, intestinal barrier function

## Abstract

This study was conducted to evaluate the effects of *Clostridium butyricum* dietary supplementation on the growth, antioxidant, immune response, gut microbiota, and intestinal barrier function of broilers under high stocking density (HSD) stress. A total of 324 1-day-old Arbor Acres male broilers were randomly assigned to three treatments with six replicates, each replicate including 18 chickens (18 birds/m^2^). The experiment lasted 6 weeks. The three treatments were basal diet (control, CON), basal diet supplemented with 1 × 10^9^ colony forming units (cfu)/kg *C. butyricum* (CB), and basal diet supplemented with 10 mg/kg virginiamycin (antibiotic, ANT). The results showed that the body weight (BW) and average daily gain (ADG) of broilers in the CB group were significantly higher than those in the CON group in three periods (*p* < 0.05). The total antioxidant capacity (T-AOC) and the superoxide dismutase (SOD) and glutathione peroxidase (GSH-Px) activity in serum of the CB group were significantly increased compared with those in the CON and ANT groups at 42 days (*p* < 0.05). At 42 days, the serum immunoglobulin M (IgM) and immunoglobulin G (IgG) levels of the CB group were significantly higher than those of the CON group. Compared with the CON group, interleukin-1β (IL-1β) in the CB group was significantly decreased in the starter and grower stages (*p* < 0.05), but there was no significant difference between the two treatment groups (*p* > 0.05). *C. butyricum* significantly decreased the high stocking density-induced expression levels of IL-1β and tumor necrosis factor-α (TNF-α) in the ileum of broilers at different stages. Additionally, *C. butyricum* could increase the expressions of claudin-1 and zonula occludens-1 (ZO-1) in intestinal tissue. Moreover, *C. butyricum* significantly increased the Sobs and Shannon indices in the CB group compared with the ANT group (*p* < 0.05), while the Ace index in the CB group was significantly higher than that of the CON group (*p* < 0.05). Furthermore, by using 16S rRNA gene sequencing, the proportion of *Bacteroides* in the CB group was increased compared to those in the CON and ANT groups at the genus level. In conclusion, *C. butyricum* supplemented into feed could improve the growth performance and feed utilization of broilers by promoting immune and intestinal barrier function and benefiting the cecal microflora.

## Introduction

During the past decade, the poultry industry in China has been developing rapidly, especially under the impact of African swine fever, when the pork production had been struck largely and the price was beyond the reach of ordinary people. Thus, considering the high price of pork, Chinese consumers were willing to offset the lower pork consumption with chicken meat. It is estimated that, in 2020, the annual sales volume of broilers in China was nearly 9.34 billion, which increased by 4.5% compared with that in 2019, indicating that people’s demand for chicken is increasing (Xin et al., 2021).

To meet such high demand, the poultry industry has turned the breeding mode into a high-density, intensive, and rapid one to improve the feeding efficiency and reduce the cost (Hong et al., 2012) in order to achieve a satisfactory economic return. However, such feeding conditions may induce chronic stress, which leads to birds being more susceptible to infectious diseases, causing related physiological and immunological stress (Li W. et al., 2019) and impairing their health, thus deceasing their production efficiency. As a result, antibiotics have been widely used in the poultry industry, less than the therapeutic doses of antibiotics added in feed that can prevent common diseases and promote productive performance (Wang et al., 2016; Ben et al., 2017). However, the long-term use of antibiotics will lead to the decline of immunity (Wu et al., 2010), the presence of antibiotic residues (Pal et al., 2016), and the development of drug-resistant bacteria (Nayak and Kenney, 2002), as well as the imbalance of the intestinal flora (Allen et al., 2014). In addition, the use of antimicrobial growth promoters (AGPs) in animal feed has been banned by the Chinese government from July 01, 2020. Alternatively, the use of probiotic bacteria can improve the immune function, inhibit pathogenic microorganisms, modulate the intestinal barrier function, optimize the intestinal flora structure, etc. (Liu X. et al., 2019; Peng et al., 2019; Zhan et al., 2019; Zhao et al., 2020).

As is well known, the microflora in the gastrointestinal tract plays an important role in poultry. Among the digestive tracts, the ceca have received the most attention because their microbiota are very diverse (Gong et al., 2002). It has been documented that cecal microbiota can protect chickens against bacterial infection (Zhu et al., 2002). *Clostridium butyricum* is a butyric acid-producing, endospore-forming, Gram-positive anaerobe bacterium that commonly exists in the intestine and feces of humans and animals (Finegold et al., 1983; Juan et al., 2017). Additionally, *C. butyricum* has stronger tolerance to lower pH, relatively higher bile concentrations, and higher temperature compared with *Lactobacillus* and *Bifidobacterium* (Kong et al., 2011; Zhang et al., 2016). Preliminary studies demonstrated that the addition of *C. butyricum* in the diet could promote growth performance, improve the immune function, and maintain or restore the intestinal microflora in broiler chickens (Meimandipour et al., 2010; Gao et al., 2012; Yang et al., 2012). In addition, *C. butyricum* could repair the intestinal barrier and increase the content of volatile fatty acids (VFAs) in the intestinal digests of broilers (Han et al., 2018; Huang et al., 2019). Thus, *C. butyricum* has been regarded as a good and safe food additive.

Although there are several studies on *C. butyricum* in broilers, its effect on broilers with high stocking density (HSD) remains to be elucidated. Therefore, the purpose of this study was to investigate the effects of *C. butyricum* on the intestinal flora, immune response, and the genes related to intestinal barrier function under HSD stress in chickens.

## Materials and Methods

### Ethics Statement

This project was approved and conducted under the supervision of the Henan Academy of Agricultural Sciences Animal Care and Use Committee. We have adopted the Animal Care and Use Guidelines governing all animal use in the experimental procedures (approval no. LLSC410006). All efforts were made to minimize suffering.

### Birds, Diets, and Housing Conditions

The study was conducted at the Institute of Animal Husbandry and Veterinary Science, Henan Academy of Agricultural Sciences (Zhengzhou), from June to August 2020. A total of 324 1-day-old Arbor Acres male broilers (Wellhope Agri-Tech Joint Stock Co. Ltd., Kaifeng, China), with similar performance, were randomly allocated to three treatment groups, each of which included six replicates of 18 chickens (18/m^2^). The experiment lasted 6 weeks.

Birds were offered the same corn–soybean basal diet, which was formulated to meet the nutrient requirements recommended by National Research Council (1994). The composition of the basal diet is shown in Table 1. The experiment consisted of three groups: the control (CON) group was supplied with basal diet, the *C. butyricum* treatment (CB) probiotic group consisted of birds fed a basal diet containing 1 × 10^9^ colony forming units (cfu)/kg *C. butyricum*, and the antibiotic treatment (ANT) group was fed a diet supplemented with 10 mg/kg virginiamycin. The *C. butyricum* supplement was purchased from Zhejiang Huijia Bio-technology Co., Ltd. (Huzhou, China) and previously determined to contain at least 3 × 10^9^ cfu/g powder. *C. butyricum* was first mixed with premix and then mixed with the other ingredients, which was in accordance with the progressive enlargement method. The diets were packed in sealed plastic bags and stored in a dry and well-ventilated storeroom until use.

**TABLE 1:** Composition and nutrient levels of basal diets (dry basis).

Items	Content	
	1–21 days old	22–42 days old
**Ingredients (%)**		
Corn	57.30	61.50
Soybean meal	37.80	33.22
Soybean oil	1.90	2.50
NaCl	0.30	0.30
Limestone	1.65	1.58
Choline chloride	0.10	0.10
CaHPO_4_	0.44	0.36
Vitamin premix^a^	0.02	0.02
Trace element premix^b^	0.20	0.20
L-lysine	0.08	0.10
DL-methionine	0.19	0.10
Phytase	0.02	0.02
Total	100.00	100.00
**Approximate composition (%)**		
Metabolic energy (MJ/kg)	12.57	12.99
Crude protein	21.50	20.05
Calcium	1.02	0.91
Available phosphorus	0.46	0.41
Lysine	1.15	1.00
Methionine	0.52	0.42
Methionine + cystine	0.85	0.77
Threonine	0.88	0.81
Tryptophan	0.29	0.27

*^a^Vitamin premix provided the following per kilogram of diet: VA = 12,000 IU, VD_3_ = 2,000 IU, VE = 20.75 mg, VK_3_ = 2.65 mg, VB_1_ = 2 mg, VB_2_ = 5 mg, VB_6_ = 2 mg, VB_12_ = 0.025 mg, biotin = 0.0325 mg, folic acid = 1.25 mg, D-pantothenic acid = 12 mg, and niacin = 50 mg.*

*^b^Trace mineral premix provided the following per kilogram of diet: Mn = 100 mg, I = 0.35 mg, Se = 0.15 mg, Zn = 75 mg, Cu = 8 mg, Fe = 80 mg, and Co = 0.2 mg.*

The birds were reared in 6 three-tiered battery cages with wire floors. The length, width, and height of each cage were 1.0, 1.0, and 0.55 m, respectively. The mesh of the floor measuring 1.5 cm × 1.0 cm (inner diameter) is commercially used in China. Throughout the experiment, feed and water were provided *ad libitum*. Chicks were exposed to continuous light during the first 2 days of age and then exposed to light for 23 h, followed by an hour of darkness per day thereafter. Ventilation was controlled to minimize contaminants such as dander, H_2_S, CO_2_, and ammonia in the house. The ambient temperature and relative humidity in the chambers were 35°C and 70% on the day of arrival, which were gradually decreased to a constant temperature and relative humidity of 24°C and 60%, respectively. All the management procedures followed commercial settings, including the normal immunization and disinfection and normal commercial feeding management.

### Sample Preparation and Parameter Measurement

The body weight (BW) and feed consumption of the broilers were recorded at 1, 21, and 42 days of age. The values of average daily feed intake (ADFI), average daily gain (ADG), and feed conversion ratio (FCR) were calculated by recording the feed intake and BW of birds in each cage during the experimental period. Mortality was checked daily, and the weights of dead broilers were used to adjust the FCR.

On days 21 and 42, two 12-h-fasted broilers from each cage were randomly selected from each group. A 4-ml blood sample was obtained from a wing vein and placed into two tubes (2 ml in each tube). Care was taken to ensure that the elapsed time between catching a bird and collecting the blood sample did not exceed 60 s. The blood samples were obliquely placed in a 37°C environment for 30 min, and then serum samples were separated by centrifugation at 3,500 × *g* for 15 min at 4°C and stored at −80°C for the immune function and antioxidant analyses. The total antioxidant capacity (T-AOC), the superoxide dismutase (SOD), glutathione peroxidase (GSH-Px), and catalase (CAT) activity, and the malondialdehyde (MDA) content in the plasma were determined with clinical chemistry assay kits (Nanjing Jiancheng Bioengineering Institute, Nanjing, China) according to the manufacturer’s instructions. The immune response status was estimated by measuring the levels of immunoglobulin A (IgA), IgG, IgM, tumor necrosis factor-α (TNF-α), and interleukin-1β (IL-1β) in serum and secretory immunoglobulin A (sIgA) in ileum. These indices were measured using chicken-specific ELISA kits (Nanjing Jiancheng Bioengineering Institute, Nanjing, China) according to the manufacturer’s instructions.

The broilers were euthanized by vein injection of 3% sodium pentobarbital (25 mg/kg BW; Sigma, St. Louis, MO, United States), followed by immediate dissection. The thymus, spleen, and bursa were immediately collected and weighed, and the absolute and relative weights (grams of organ per kilogram BW) were used for statistical analysis. The ceca were removed aseptically, clamped with forceps, and placed in sterile plastic bags on ice, and then the cecal content samples were collected into 2-ml sterilized tubes, immediately frozen in liquid nitrogen, and stored at −80°C until analysis. The middle of the ileum was isolated and split longitudinally on ice, and then the intestinal segment was washed by pre-cooled sterile normal saline. The mucous layer attached to the ileum wall was gently scraped off with a small sterile spatula into a 2-ml sterile tube and stored in liquid nitrogen for mRNA analysis.

Total RNA was extracted from ileal mucous membrane samples using TaKaRa MiniBEST Universal RNA Extraction Kit (TaKaRa, Beijing, China) according to the manufacturer’s instructions (Zhao K. et al., 2018). DNase I was used during the RNA isolation process to exclude contamination with genomic DNA. Then, RNA was solubilized in RNase-free water. The quantity and quality of the purified RNA were determined by measuring the absorbance at 260/280 nm using a NanoDrop^®^ ND-1000 spectrophotometer (Thermo Scientific, Waltham, MA, United States), and the RNA integrity was assessed by agarose gel electrophoresis. Only RNA samples that had an OD_260/280_ between 1.8 and 2.0 and had good integrity were used for the subsequent cDNA synthesis using the PrimeScript™ RT Master Mix Kit (TaKaRa, Beijing, China) following the manufacturer’s instructions.

Real-time PCR was conducted using the TB Green^®^ Premix Ex Taq™ II (TaKaRa, Beijing, China) in a Light Cycler^®^ 96 (Roche, Basel, Switzerland) (Zhao K. et al., 2018). Amplification was performed in a total volume of 20 μl, containing 0.8 μl of each primer (10 mM), 2 μl of the threefold diluted cDNA, 10 μl of the SYBR^®^ Premix Ex Taq™ II, and 6.4 μl of sterilized double-distilled water. The real-time PCR program was as follows: 95°C denaturation for 30 s, 95°C denaturation for 5 s, and 60°C annealing and extension for 20 s, 40 cycles. At the end of each PCR, a dissociation curve of 0.5°C increases from 65 to 95°C was plotted to confirm the single amplification in each reaction. Each sample was run in triplicate, and reactions without templates were used as negative controls.

The *GAPDH* reference gene was chosen for the relative expressions of the target genes. The relative expression of mRNA was calculated using the 2^–ΔΔCT^ method (Livak and Schmittgen, 2001). The primers used in this study are listed in Table 2.

**TABLE 2:** Primer sequences of the target and reference genes.

Genes	Primer sequence (5′–3′)	Accession no.
*GAPDH*	F: TGGTGCTAAGCGTGTTATCATCT R: GGCATGGACAGTGGTCATAAGAC	NM_204305.1
*TNF-*α	F: GAGCGTTGACTTGGCTGTC R: AAGCAACAACCAGCTATGCAC	NM_204267
*IL-1*β	F: ACTGGGCATCAAGGGCTA R: GGTAGAAGATGAAGCGGGTC	NM_204524
*IL-10*	F: GCTGTCACCGCTTCTTCACCT R: GGCTCACTTCCTCCTCCTCATC	EF554720.1
*NF-*κ*B*	F: GTGTGAAGAAACGGGAACTG R: GGCACGGTTGTCATAGATGG	NM_205129
*Claudin-1*	F: CATACTCCTGGGTCTGGTTGGT F: CATACTCCTGGGTCTGGTTGGT	AY750897.1
*Occludin-1*	F: ACGGCAGCACCTACCTCAA R: GGGCGAAGAAGCAGATGAG	D21837.1
*ZO-1*	F: CTTCAGGTGTTTCTCTTCCTCCTC R: CTGTGGTTTCATGGCTGGATC	XM_413773

*GAPDH, glyceraldehyde-3-phosphate dehydrogenase; TNF-α, tumor necrosis factor-α; IL, Interleukin; NF-κB, nuclear factor kappa-B; ZO-1, zonula occludens-1.*

### 16S rRNA Gene Amplification of the V3 + V4 Regions, Sequencing, and Bioinformatics Analysis

The 16S rRNA gene amplicons were used to determine the diversity and structural comparisons of the bacterial species in cecal contents using the Illumina MiSeq platform (Illumina, San Diego, CA, United States) according to the standard protocols by Majorbio Bio-Pharm Technology Co. Ltd. (Shanghai, China). Microbial community genomic DNA was extracted from the cecal contents using the E.Z.N.A.^®^ soil DNA Kit (Omega Bio-tek, Norcross, GA, United States) according to the manufacturer’s instructions. The hypervariable regions V3 + V4 of the bacterial 16S rRNA gene were amplified with the primer pairs 338F (5′-ACTCCTACGGGAGGCAGCAG-3′) and 806R (5′-GGACTACHVGGGTWTCTAAT-3′). Raw 16S rRNA gene sequencing reads were demultiplexed, quality filtered by Trimmomatic, and merged by FLASH. Operational taxonomic units (OTUs) with a 97% similarity cutoff were clustered using UPARSE (version 7.1^1^), and chimeric sequences were identified and removed. The taxonomy of each OTU representative sequence was analyzed using the RDP Classifier^2^ against the 16S rRNA database (Silva SSU128) using a confidence threshold of 0.7.

The diversity, composition, and difference of the bacterial communities were analyzed on the I-Sanger Cloud Platform^3^, which was provided by Majorbio Bio-Pharm Technology Co. Ltd. (Shanghai, China) (Wang et al., 2020). Data on OTU abundance were normalized using a standard sequence number corresponding to the sample with the least number of sequences. The diversity of the bacterial communities was determined by alpha diversity analyses. The Chao1 and ACE indices simply refer to the number of species in the community, regardless of the abundance of each species in the community; the Shannon’s diversity index considers both richness and evenness. Taxonomic composition was investigated at the phylum and genus levels. Differences in the microbiota composition from phylum to genus were analyzed using the linear discriminant analysis (LDA) effect size (LEfSe) method (Wang et al., 2020).

### Statistical Analysis

The experiment was conducted as a randomized complete block design with six replicates. Each pen was considered an experimental unit. The data followed normal distribution and were analyzed by one-way analysis of variance (ANOVA) using SPSS statistical software (ver. 20.0 for Windows, SPSS, Inc., Chicago, IL, United States). Tukey’s multiple range test was used to determine the means and differences among treatments. The α-diversity analysis was based on the Mann–Whitney *U* test. Significant distinction of the microbial communities was estimated by the Kruskal–Wallis *H* test. The different abundance rates of the microbiota communities from phylum to genus were analyzed using the LEfSe algorithm. The LDA score (>2) was used to examine the significant microbiota communities. Data are presented as the mean ± SE of six replicates. Significant differences among treatments were determined at *p* < 0.05.

## Results

### Growth Performance and Feed Utilization

The effects of *C. butyricum* on the growth performance and feed utilization of the broilers are shown in Table 3. The BW and ADG of broilers in the CB and ANT groups were significantly higher than those in the CON group at periods of 1–21 and 1–42 days (*p* < 0.05). The FCRs in the CB and ANT groups were significantly lower than that in the CON group at periods of 1–21 and 1–42 days (*p* < 0.05). The ADFI of broilers among all groups was not affected by treatment in this experiment (*p* > 0.05).

**TABLE 3:** Effects of *Clostridium butyricum* on the growth performance and feed utilization of broilers^1^.

Items	CON	CB	ANT	*p*-value
Initial BW	44.14 ± 0.17	44.82 ± 0.20	44.23 ± 0.30	0.107
**1–21 days**				
BW (g)	739.24 ± 6.49b	770.21 ± 7.63a	785.35 ± 4.14a	<0.001
ADG (g/day)	33.07 ± 0.28b	34.47 ± 0.35a	35.27 ± 0.20a	<0.001
ADFI (g/day)	43.62 ± 0.49	43.08 ± 0.21	44.33 ± 0.80	0.307
FCR (g/g)	1.32 ± 0.02a	1.25 ± 0.01b	1.26 ± 0.02b	0.010
**1–42 days**				
ADG (g/day)	48.03 ± 1.86b	51.06 ± 1.08a	52.08 ± 0.43a	<0.001
ADFI (g/day)	84.90 ± 3.09	87.19 ± 1.33	87.35 ± 1.90	0.139
FCR (g/g)	1.83 ± 0.01a	1.77 ± 0.01b	1.74 ± 0.01b	<0.001

*^1^Mean values within the same row without similar lowercase letters are significantly different (p < 0.05). Data are presented as the means ± SE of six replicates.*

*CON, no additives; CB, basal diet supplemented with 1 × 10^9^ cfu C. butyricum/kg; ANT, basal diet supplemented with 10 mg/kg virginiamycin; BW, body weight; ADG, average daily gain; ADFI, average daily feed intake; FCR, feed conversion ratio.*

### Lymphoid Organs

As shown in Table 4, the bursa index in the CB and ANT groups was significantly lower than that in the CON group (*p* < 0.05) at 21 days. At 42 days of age, the spleen index of the CB group and the ANT group was significantly higher than that of the CON group (*p* < 0.05). There was no significant difference in the thymus index among the groups in the starter and grower stages (*p* > 0.05).

**TABLE 4:** Effects of *Clostridium butyricum* on the immune organ indices of broilers^1^.

Items (g/kg)	CON	CB	ANT	*p*-value
**21 days**				
Spleen index	0.85 ± 0.09	0.83 ± 0.05	0.86 ± 0.08	0.958
Thymus index	3.30 ± 0.23	3.37 ± 0.15	3.59 ± 0.36	0.262
Bursa index	2.33 ± 0.07a	1.98 ± 0.09b	1.97 ± 0.17b	0.032
**42 days**				
Spleen index	1.31 ± 0.03b	1.40 ± 0.02a	1.41 ± 0.02a	0.022
Thymus index	2.87 ± 0.20	2.89 ± 0.18	2.95 ± 0.13	0.942
Bursa of index	1.24 ± 0.12	1.23 ± 0.10	1.25 ± 0.06	0.985

*^1^Mean values within the same row without similar lowercase letters are significantly different (p < 0.05). Data are presented as the mean ± SE of six replicates.*

*CON, no additives; CB, basal diet supplemented with 1 × 10^9^ cfu C. butyricum/kg; ANT, basal diet supplemented with 10 mg/kg virginiamycin.*

### Antioxidation

The effects of *C.* butyricum on the stress indicators of the broilers are presented in Table 5. *C. butyricum* and antibiotic treatments were found to significantly affect the levels of MDA and the activity of GSH-Px (*p* < 0.05) in serum, but no significant differences were found for T-AOC, SOD, and CAT at 21 days (*p* > 0.05). The T-AOC, SOD, and GSH-Px in serum of the CB group were significantly increased compared with those in the CON and ANT groups at 42 days (*p* < 0.05). The serum MDA content in the CB group had the lowest value and was significantly lower than those in the other two groups (*p* < 0.05). There was no significant difference in serum CAT activity among all groups (*p* > 0.05).

**TABLE 5:** Effects of *Clostridium butyricum* on the serum antioxidant indices of broilers^1^.

Items	CON	CB	ANT	*p*-value
**21 days**				
T-AOC (U/ml)	0.64 ± 0.05	0.69 ± 0.06	0.66 ± 0.04	0.729
SOD (U/ml)	104.93 ± 7.42	114.27 ± 12.00	117.83 ± 7.87	0.632
MDA (nmol/ml)	2.81 ± 0.06a	2.34 ± 0.04b	2.59 ± 0.09ab	0.010
CAT (U/ml)	8.52 ± 0.29	8.03 ± 0.13	8.39 ± 0.34	0.433
GSH-Px (U/ml)	1,031.87 ± 40.14b	1,247.54 ± 38.68a	986.48 ± 29.95b	0.010
**42 days**				
T-AOC (U/ml)	0.72 ± 0.08b	0.98 ± 0.07a	0.78 ± 0.02b	0.018
SOD (U/ml)	110.98 ± 6.81b	133.99 ± 4.51a	117.57 ± 4.50b	0.024
MDA (nmol/ml)	3.17 ± 0.09a	2.77 ± 0.06b	3.04 ± 0.06a	0.005
CAT (U/ml)	8.27 ± 0.31	8.96 ± 0.43	8.91 ± 0.39	0.387
GSH-Px (U/ml)	1,267.23 ± 39.88b	1,461.22 ± 41.32a	1,301.45 ± 59.37b	0.013

*^1^Mean values within the same row without similar lowercase letters are significantly different (p < 0.05). Data are presented as the mean ± SE of six replicates.*

*CON, no additives; CB, basal diet supplemented with 1 × 10^9^ cfu C. butyricum/kg; ANT, basal diet supplemented with 10 mg/kg virginiamycin; T-AOC, total antioxidant capacity; SOD, superoxide dismutase; MDA, malondialdehyde; CAT, catalase; GSH-Px, glutathione peroxidase.*

### Immune Indices and Inflammatory Factors

**FIGURE 1:**
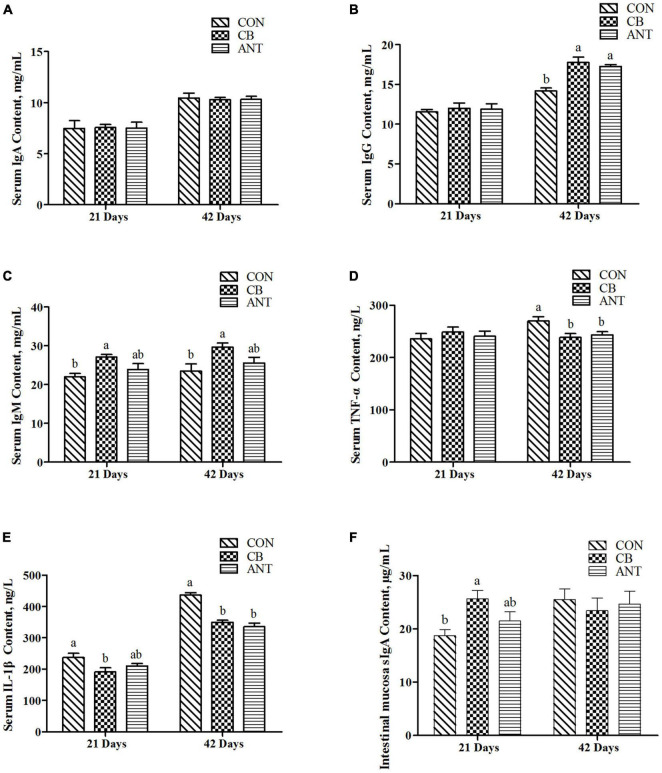
Effects of *Clostridium butyricum* on the immune indices and inflammatory factors of broilers. **(A–C)** Serum immunoglobulin concentrations in broilers. **(D,E)** Serum inflammatory factor levels in broilers. **(F)** Intestinal secretory immunoglobulin A level in broilers. The *bars* represent the mean ± SE (*n* = 6/group). *Different letters over the bars* indicate statistically significant differences between the groups (*p* < 0.05); the *same letters over the bars* indicate no statistically significant differences between the groups (*p* > 0.05). CON, no additives; CB, basal diet supplemented with 1 × 10^9^ cfu *C. butyricum*/kg; ANT, basal diet supplemented with 10 mg/kg virginiamycin.

As shown in Figure 1, the serum IgA level was not affected by the treatment at 21 and 42 days (*p* > 0.05). The IgM content had the highest value in the starter and grower stages and was significantly higher than that in the CON group (*p* < 0.05). At 42 days, the serum IgG level of the CON group was significantly lower than those of the two experimental groups (*p* < 0.05). The level of TNF-α in the CB group was significantly lower than those in the CON group and the antibiotic added group. Compared with the CON group, IL-1β in the CB group was significantly decreased in the starter and grower stages (*p* < 0.05), but there was no significant difference between the two treatment groups (*p* > 0.05).

At the starter stage of the experiment, the level of sIgA in the CB group had the highest value, which was significantly higher than that in the CON group, but had no significant difference from that in the ANT group.

### Expressions of Intestinal Immune Response and Barrier Function Genes

**FIGURE 2:**
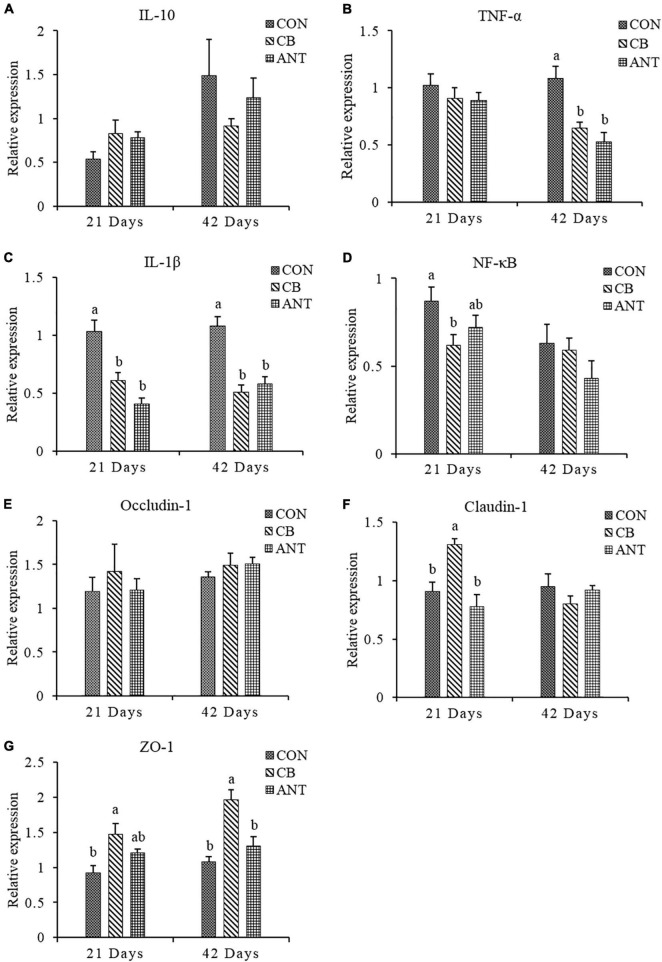
Relative expressions of the immune and intestinal mucosa barrier-related genes in the ileum of broilers. **(A–D)** mRNA expression levels of ileal cytokines in broilers. **(E–G)** mRNA expressions levels of ileal tight junction proteins in broilers. The *bars* represent the mean ± SE (*n* = 6/group). *Different letters over the bars* indicate statistically significant differences between the groups (*p* < 0.05); the *same letters over the bars* indicate no statistically significant differences between the groups (*p* > 0.05). CON, no additives; CB, basal diet supplemented with 1 × 10^9^ cfu *C. butyricum*/kg; ANT, basal diet supplemented with 10 mg/kg virginiamycin.

The expressions of the immune and intestinal barrier-related genes detected in the different groups are shown in Figure 2. The expression level of *TNF-*α in the ileum was significantly decreased in the CB and ANT groups compared with that in the CON group at 42 days (*p* < 0.05). The expression level of IL-1β in the CON group was significantly higher than those in the two treatment groups at 21 and 42 days (*p* < 0.05). The expression level of *NF-*κ*B* in the CB group was significantly lower than that in the CON group, but had no significant difference from that in the ANT group at 21 days. Compared with that in the other two groups, *C. butyricum* supplementation induced the highest expression of *Claudin-1* in the CB group at 21 days (*p* < 0.05). At the grower stage, there was no significant difference in the levels of *Claudin-1* among all groups (*p* > 0.05). Compared with that in the CON group, the expression level of zonula occludens-1 (*ZO-1*) in the CB group was significantly elevated in the starter and grower stages (*p* < 0.05), while there was no significant difference between the CON and ANT groups (*p* > 0.05). There were no significant differences in the relative expressions of the interleukin-10 (*IL-10*) and *Occludin-1* genes of the groups at different stages (*p* > 0.05).

### Cecal Microbial Analysis

**FIGURE 3:**
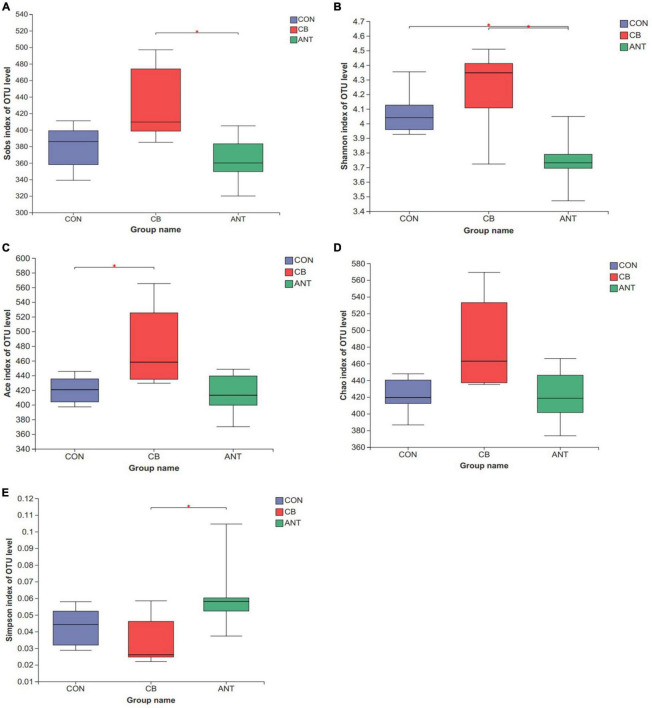
Diversity of the bacterial communities. The alpha diversity indices of the bacterial communities were determined by observed richness (Sobs) **(A)**, Shannon diversity index (Shannon) **(B)**, ACE estimator (Ace) **(C)**, Chao 1 estimator (Chao) **(D)**, and the Simpson estimator **(E)**. The *bars* represent the mean ± SE (*n* = 6/group). *Asterisk over the bars* indicates statistically significant differences between the groups (*p* < 0.05). CON, no additives; CB, basal diet supplemented with 1 × 10^9^ cfu *C. butyricum*/kg; ANT, basal diet supplemented with 10 mg/kg virginiamycin.

The alpha diversity indices, including Sobs, Shannon, Ace, Chao, and Simpson, of the intestinal bacterial community were analyzed among the different groups using the Wilcoxon rank-sum test (Figures 3A–E). *C. butyricum* significantly increased the Sobs and Shannon indices in the CB group compared with the ANT group (*p* < 0.05), while the reverse tendency was found for the Simpson index (*p* < 0.05). The Ace index in the CB group was significantly higher than that in the CON group (*p* < 0.05). There was no significant difference in the Chao index among all groups (*p* > 0.05).

**FIGURE 4:**
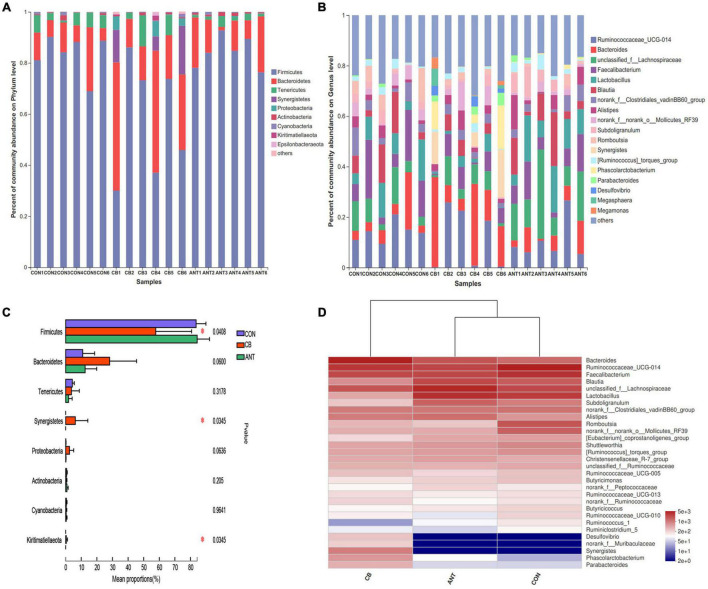
Composition of cecal microbiota and differential species of broilers identified at the phylum and genus levels. **(A)** Relative abundance of the most abundant bacterial phyla. **(B)** Relative abundance of the most abundant bacterial genus. **(C)** Relative abundance difference analysis of cecum bacterial species of broilers at the phylum level. **(D)** Heatmap of the top 30 relatively abundant genera of the microbiota communities. The *bars* represent the mean ± SE (*n* = 6/group). *Asterisk over the bars* indicates statistically significant differences between the groups (*p* < 0.05). CON, no additives; CB, basal diet supplemented with 1 × 10^9^ cfu *C. butyricum*/kg; ANT, basal diet supplemented with 10 mg/kg virginiamycin.

The composition of cecal microflora is presented in Figures 4A–D. At the phylum level, Firmicutes, Bacteroidetes, and Tenericutes were the three most abundant bacterial phyla in all samples (Figure 4A). The genera *Ruminococcaceae*_UCG-014, *Bacteroides*, unclassified_f__*Lachnospiraceae*, *Faecalibacterium*, and *Lactobacillus* were the most prevalent in all of the groups (Figure 4B). The relative abundance of *Firmicutes* was decreased and Synergistetes and Kiritimatiellaeota were raised in the CB group compared to those in the CON and ANT groups (*p* < 0.05) (Figure 4C). At the genus level, the heatmap (Figure 4D) of the top 30 most relatively abundant genera showed the cecal microbial communities of broilers fed the basal and antibiotic diets forming a common cluster, while those of broilers fed the *C. butyricum* diet structured a separate cluster, indicating that the microbial composition was similar between the CON and ANT groups.

**FIGURE 5:**
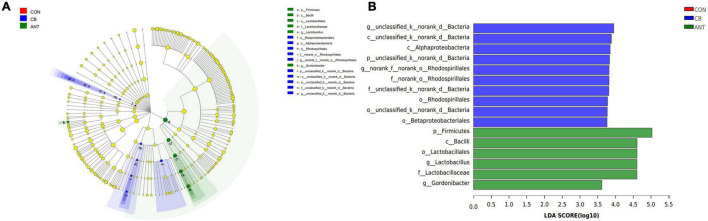
Linear discriminant analysis (LDA) effect size (LEfSe) was used to calculate the most significantly abundant cecal microbiota of broilers. **(A)** Cladogram measured from the LEfSe analysis. **(B)** LDA scores generated for the differentially abundant microbiota (LDA > 2, *p* < 0.05).

Furthermore, LEfSe analysis (Figures 5A,B) was performed to identify the significant taxa in phylotypes. In the aggregate, 10 genera were detected with a LDA threshold > 2. Broilers that were fed *C. butyricum* had significantly enriched (*p* < 0.05) Alphaproteobacteria and unclassified_k_norank_d_*Bacteria* relative abundance at the class level. The relative abundance of norank_o_*Rhodospirillales* and unclassified_k_norank_d_*Bacteria* in the CB group was increased (*p* < 0.05) compared with that in the CON and ANT groups at the family level.

## Discussion

In the past decade, the effects of probiotics have been widely studied. A number of recent studies have confirmed that probiotics can promote nutrient utilization efficiency and improve growth performance in animals (Liao X. D. et al., 2015; Abdel-Latif et al., 2018; Zhao X. et al., 2018; Li H. et al., 2019; Wang et al., 2019a). In the present study, broilers fed diets supplemented with *C. butyricum* had higher BW and ADG and lower FCR compared with those in the CON group, and no differences were found in the growth and feed utilization between the CB group and the ANT group. Similar results have been reported in Svejstil et al. (2019), which showed that a *C. butyricum* CBM 588-supplemented diet positively affected the growth and feed conversion of broiler chickens. Zhao X. et al. (2018) found that *C. butyricum* supplementation at 1 × 10^9^ cfu/kg could improve the growth performance of broilers, and Liao X. D. et al. (2015) reported that the ADG of chickens fed with *C. butyricum* was higher than that of the control group. Moreover, Zhang et al. (2014, 2016) also documented that supplementation of *C. butyricum* achieved similar effects to the antibiotic group in promoting the growth performance of *Escherichia coli* K88-challenged broiler chickens. Thus, the current study and the above research works indicated that the supplementation of *C. butyricum* in the diet could promote broiler growth and be used as a substitute for antibiotics. The mechanism by which *C. butyricum* promotes growth performance may be partly attributed to its ability to ameliorate the intestinal microecological environment *via* improving the gastrointestinal digestive enzyme activity and producing short-chain fatty acids, which are an energy source for animals and have a proliferative effect on colonocytes (Topping and Clifton, 2001; Nakanishi et al., 2003; Zhang et al., 2016). However, Zhang et al. (2011) and Han et al. (2018) found that the addition of *C. butyricum* to the diet had no effect on the growth performance and feed utilization of broilers. These inconsistent results may be attributed to the different basal diets, broiler breeds, administration levels, and growth phases.

As is well known, antioxidant enzymes play vital roles in antioxidant defense mechanisms (Seven et al., 2009). In the current study, the results showed that dietary supplementation with *C. butyricum* could increase serum SOD and GSH-Px activity, whereas it decreased the MDA concentration in the serum of broilers. Our data corresponded with the findings of Liao X. et al. (2015) and Zhan et al. (2019), which showed that broilers and hens fed with *C. butyricum* increased the SOD, GSH-Px, and CAT concentrations and decreased the MDA level in serum. Moreover, Liao X. D. et al. (2015) and Duan et al. (2017a) reported that the activity of antioxidant enzymes in the intestine could be enhanced by *C. butyricum* addition in the diet of broilers and kuruma shrimp. This may be due to *C. butyricum* being able to produce butyrate and H_2_, which have been proven to regulate oxidative stress by reducing reactive oxygen metabolites and increasing antioxidant enzyme activity (Liao X. D. et al., 2015). Furthermore, Duan et al. (2017a,b) indicated that *C. butyricum* supplementation in the diet could decrease the O^2–^ generation capacity and improve stress tolerance. Taken together, it could be suggested that the supplementation of *C. butyricum* has beneficial effects on the regulation of oxidative stress by improving the activity of antioxidant enzymes in broilers.

As is well known, immunoglobulins and cytokines are commonly used to evaluate the immune status of birds because both play an important role in immune function (Zhan et al., 2019). Numerous studies have demonstrated that dietary supplementation with *C. butyricum* has beneficial effects on the immune function in livestock and poultry. To illustrate, diets with *C. butyricum* increased the concentrations of IgA and IgM and decreased the pro-inflammatory factors TNF-α and IL-1β in the serum of weaned piglets (Wang et al., 2019a). Similarly, Wang et al. (2019b) reported that the level of immunoglobulin in piglets fed a diet with probiotics increased significantly at 14 and 28 days. In the present study, the serum IgM and IgG contents were significantly higher than that in the CON group, and the levels of TNF-α and IL-1β in the CB group were lower than those in the CON group and antibiotic added group at 42 days. Similar results were observed in Yang et al. (2012) and Zhan et al. (2019), who found that birds receiving diets with *C. butyricum* promoted the concentrations of serum IgG and IgM. Liao X. D. et al. (2015) and Han et al. (2018) reported that the addition of *C. butyricum* in broiler diet could increase the content of IgM in serum compared with the control group. In addition, in the intestinal immunological barrier, mucosal sIgA is the predominant intestinal immunoglobulin, which is an important defense against various endogenous/exogenous microorganisms and other antigens. Liu L. et al. (2019) provided evidence that supplementation with *C. butyricum* accelerated sIgA secretion in the small intestine, especially in the duodenum of weaning rex rabbits. Zhang et al. (2014) reported that broilers fed with *C. butyricum* had greater jejunum mucosal sIgA concentrations than the other treatment groups. In accordance with previous findings, our data showed that supplementation with *C. butyricum* can enhance the production of sIgA in the starter stage. The spleen is a vital lymphoid organ in broilers. In the present study, the spleen index decreased in the CON group. This may be attributed to the HSD in this group. High densities can lead to more conflict, stress, and immunocompromise in birds (Li W. et al., 2019). Thus, a lower spleen index is associated with an increased level of physiological stress. As mentioned above, *C. butyricum* has indirect favorable effects on antioxidant capacity; as a result, the spleen index was higher in the CB group than that in the group without *C. butyricum* supplementation. This is consistent with current research. Zhan et al. (2019) found that laying hens fed with 5 × 10^4^ cfu/g *C. butyricum* had a higher spleen index compared with those in the control group. Additionally, Chen et al. (2013) reported that broilers given 1 × 10^9^ cfu/g *C. butyricum* increased the relative weights of the spleen and the bursa of Fabricius compared to those not given treatment. Collectively, these aforementioned results demonstrated that the addition of *C. butyricum* to the diet effectively improved the immune function and alleviated the inflammatory response of broilers. This may be attributed to *C. butyricum* being able to act as a mitogen, leading to the proliferation and transformation of B cells, which would mature after homing and class switching to immunoglobulin secretion (Murayama et al., 1995).

It has been reported that a HSD increased oxidative stress and induced intestinal mucosal injury in broilers (Li W. et al., 2019; Ma et al., 2020). In the present study, *C. butyricum* significantly decreased the HSD-induced expression levels of *IL-1*β and *TNF-*α in the ileum of broilers at different stages. Similar results were observed in previous studies, indicating that supplementation with *C. butyricum* significantly decreased the expression levels of pro-inflammatory cytokines in the intestinal epithelial cells of chickens after *Salmonella* infection (Zhao et al., 2020). Moreover, Zhao et al. (2017) demonstrated that treating *Salmonella*-infected broilers with *C. butyricum* could significantly reduce the expression levels of *IL-1*β, *IL-8*, and *TNF-*α in the liver, spleen, and cecum to different extents.

Furthermore, we found that *C. butyricum* could downregulate the expression of *NF-*κ*B* in the intestinal mucous membrane of broilers under HSD, which is consistent with previous studies showing that *C. butyricum* can alleviate inflammation in chickens by downregulating the *TLR4*-, *MyD88*-, and *NF-*κ*B*-dependent signaling pathways (Zhao et al., 2017, 2020). In this context, *C. butyricum* has the ability to influence the expressions of cytokines in order to enhance the host immune system and provides a beneficial role for the host by synthesizing immunosuppressive factors (Gao et al., 2012; Quanxin et al., 2013).

As is well known, tight junctions play a vital part in the intestinal mucosal barrier, which can prevent bacteria and toxins entering the blood circulation (Ballard et al., 1995). Moreover, the permeability of the tight junctions between cells determines the barrier function of the entire intestinal epithelial cell (Turner, 2006). Claudin-1, occludin-1, and ZO-1, the indices of which were evaluated in this study, are important proteins responsible for the structural and functional organization of tight junctions (Fanning et al., 1998). In the current experiment, we detected that *C. butyricum* could increase the expressions of *Claudin-1* and *ZO-1* in intestinal tissue, which is consistent with a past report showing that the mRNA level of *ZO-1* in broilers with *C. butyricum* diet was significantly higher (Zhao et al., 2020). Taken together, the results of the present study and those of some previous studies demonstrated that dietary supplementation with *C. butyricum* can improve the expressions of tight junction proteins to maintain intestinal barrier function and decrease the pro-inflammatory cytokine, IL-1β and TNF-α, expressions in order to modulate the inflammatory response (Gao et al., 2012; Chen et al., 2018; Liu L. et al., 2019).

The gut microflora is extremely complex and plays a key role in feed utilization, diversity, intestinal integrity, and animal health (Wang et al., 2019b). The results of this study showed that the alpha diversity indices of the CB group were superior compared to those of the CON and ANT groups. This indicated that *C. butyricum* supplementation could increase the diversity of the bacterial community and alter the intestinal microbial composition in broilers. Similar results have been reported in some previous studies, which indicated that *C. butyricum* benefits the diversity of intestinal bacteria (Huang et al., 2019; Zhao et al., 2020). This may be due to the ability of *C. butyricum* to inhibit the presence of opportunistic pathogens. Nakanishi and Tanaka (2010) reported that *C. butyricum* can produce a bacteriocin that can inhibit the growth of pathogenic clostridia in the intestinal tracts. On other hand, *C. butyricum* has the ability to produce VFAs, which can inhibit the growth of pathogenic bacteria by regulating the intestinal pH and promoting the growth of beneficial bacteria (Yang et al., 2012). Moreover, we found that the antibiotic-supplemented group had secondary diversity compared with the CON group, indicating that, although an antibiotic diet could facilitate the growth of chickens, the diversity and the composition of the intestinal microflora of the antibiotic-supplemented chickens were not better than those in chickens fed the basic diet.

As aforementioned, the intestinal microflora plays an important role in poultry, especially the cecum, which is the main site of fermentation in the gastrointestinal tract. The results of the present study showed that Firmicutes and Bacteroidetes were the dominant phyla of the cecum in broilers. The relative abundance of Bacteroidetes in the CB group showed a modest increase; this may have been responsible for the depletion of Firmicutes representatives in *C. butyricum* treatment. Hagihara et al. (2018) reported that the administration of *C. butyricum* MIYAIRI 588 could raise the abundance of *Bacteroides* in the gut of mice. Han et al. (2020) found that diet supplemented with *C. butyricum* improved the abundance of Bacteroidetes in the colon and indicated that Bacteroidetes was positively correlated with feed utilization in weaned piglets. In addition, it has been documented that *Bacteroides* could promote the supply efficiency of daily calories *via* improving the hydrolysis of indigestible dietary polysaccharides (Xu et al., 2003). This may partly be the reason for the diet supplemented with *C. butyricum* having superior ADG and lower feed:gain ratio (F/G) compared to the CON group in the present study. Furthermore, Bacteroidetes are involved in intestinal immunomodulatory function and can protect the intestine from pro-inflammatory reactions (Yanagibashi et al., 2013). It has been indicated that *Bacteroides* could modulate the T-cell-dependent immune responses and promote IgA production in Peyer’s patch cells (Hagihara et al., 2018). Therefore, the increased sIgA, IgM, and IgG contents and the decreased TNF-α and IL-1β expression levels in the ileum and serum due to *C. butyricum* supplementation have favorable effects on immune function, which may have partially contributed to the increased relative abundance of Bacteroidetes in cecal contents.

## Conclusion

The results of the present study demonstrated that dietary supplementation of 1 × 10^9^ cfu/kg *C. butyricum* could improve the growth performance, feed utilization, and immune function, promote the intestinal barrier function, and regulate intestinal microbes by increasing the relative abundance of Bacteroidetes. In addition, in this study, the CB and ANT groups had similar performance, so *C. butyricum* could be considered as a substitute for antibiotics. These findings provide practical support and theoretical basis for the application of *C. butyricum* in broiler production.

## Data Availability Statement

The original contributions presented in the study are publicly available. This data can be found here: PRJNA765463.

## Ethics Statement

The animal study was reviewed and approved by Henan Academy of Agricultural Sciences Animal Care and Use Committee (approval number LLSC410006). Written informed consent was obtained from the owners for the participation of their animals in this study.

## Author Contributions

WL and BX implemented the study and wrote the manuscript. LW, QS, FW, and WD assisted in conducting the experiments and collecting the samples. HM, CF, and GW were responsible for sample analysis and determination. SL supervised and provided continuous guidance for the experiments. All authors contributed to the article and approved the submitted version.

## Conflict of Interest

The authors declare that the research was conducted in the absence of any commercial or financial relationships that could be construed as a potential conflict of interest.

## Publisher’s Note

All claims expressed in this article are solely those of the authors and do not necessarily represent those of their affiliated organizations, or those of the publisher, the editors and the reviewers. Any product that may be evaluated in this article, or claim that may be made by its manufacturer, is not guaranteed or endorsed by the publisher.

## Footnotes

1
http://drive5.com/uparse/

2
http://rdp.cme.msu.edu/

3
www.i-sanger.com
